# Preliminary Finding on Anomalous Cleavage and Degeneration of Intestinal Nematode Eggs (*Nematodirus* sp.) after Oral Administration of Medium-Chain Fatty Acid in Calves

**DOI:** 10.5402/2011/616537

**Published:** 2011-04-04

**Authors:** Hiroshi Sato, Takashi Kurosawa

**Affiliations:** School of Veterinary Medicine, Rakuno-Gakuen University, Bunkyodai, Ebetsu, Hokkaido 069-8501, Japan

## Abstract

Medium-chain fatty acids (MCFAs) consisting of 8 to 12 carbons are knowns exhibit antimicrobial activity against bacteria and gut protozoa. However, little information is available on their effect in helminthes. The effect of MCFA on an intestinal nematode (*Nematodirus* sp.) was therefore evaluated in four calves (4 to 11 month old). Edible fat containing MCFA was administered into the abomasum of the calves by practical stimulation of the reticular groove reflex for 5 days, and the resulting fecal egg shedding was examined. Although MCFA had a weak effect on fecal egg number of *Nematodirus* sp., morphologically anomalous eggs were observed. Anomalies manifested as degenerated eggs with ova granulation or shell rupture, irregular monocellular egg, and disproportional cleavage at the 2-, 4-cell and subsequent stages, despite normal shedding at 8- or 16-cell stages in most cases. Thus, MCFA administration brought cleavage disturbance and degeneration of *Nematodirus* sp. eggs.

## 1. Introduction

Medium-chain fatty acids (MCFA), which are composed of 8 to 12 carbon chains (i.e., octanoic, decanoic, and dodecanoic acids), have ease with which they are absorbed and metabolized in host animals [1, 2]. The MCFA, are conventionally used as edible fat and others in triglyceride form of the constituent MCFA. After ingestion, the acids are easily released by hydrolysis and easily absorbed in the small intestine. Since the absorbed MCFA are more readily utilized as a metabolic fuel than long chain fatty acids [2], dietary and parenteral MCFA (as triglyceride in practice) are given to hospitalized humans [2, 3] and swine [1].

In addition to the nutritional benefits of the MCFA, their antibacterial [4], anticoccidial [5, 6], and ruminal defaunative [7] activities have attracted considerable interest over the last two decades. In recent years, given the stringent safety standards associated with the use of antimicrobials in both humans and livestock animals, worldwide demand has been increasing for the alternative agents. In this regard, following a finding on the anthelmintic effect of MCFA given to fish infested with a monogenean parasite [8], an evaluation of MCFA for controlling parasitic nematodes in livestock has been desired for some time. After administering MCFA into the abomasum of calves infested with *Nematodirus* sp., we observed fecal shedding of various anomalous eggs. The characteristics of the anomalous eggs are described here for the first time.

## 2. Materials and Methods

### 2.1. Animals

Four Holstein calves (2 males and 2 females; 4 to 11 months old) were grazed in the summer season on a pasture having mixed herbage without any feed supplements. Growth of the calves was retarded due to their lower plane of nutrition in the past. All the calves were infected with coccidia and nematodes, including *Nematodirus* sp.

### 2.2. Administration of Fat

The MCFA was administered to the calves in triglyceride form (Kao, Tokyo, Japan), which is commercially available as human edible fat in the food industry. The triglyceride appeared as a transparent liquid at room temperature but was solid in winter outdoor and had has a less unpleasant flavor than the free fatty acid form. The fatty acid profile of the triglyceride was analyzed by gas chromatography accompanying a flame ionization detector (Shimadzu, Kyoto, Japan). The results revealed the fatty acid content to be 33.5% octanoic acid (C_8_), 35.5% decanoic acid (C_10_), 17.6% dodecanoic acid (C_12_), and 13.4% other acids.

In milk-fed young ruminants including calves, oral liquid ingestion follows two separate routes as it enters the gastrointestinal tract; one is referred to as ruminal flow (in forestomach) and the other is via direct entry into the abomasum followed by an inflow into the small intestine. Ingested nutrients and chemicals into the rumen are frequently exposed to a microbial degradation; accordingly, not all of the ingested ones flow into the small intestine. To avoid the degradation of the given MCFA in the rumen and also to prevent the potentially harmful effects of the MCFA on rumen microbial flora [7], we attempted to administer the MCFA into the abomasum by bypassing the rumen using the reticular groove reflex, specific to neonatal ruminants. Since the reflex does not function after weaning (also in adults), this reflex was reacquired in the present calves by suckling of a warm glucose solution from a bottle fitted with a rubber teat [9]. Using this teat bottle suckling and accompanying reticular groove reflex, the MCFA (as triglyceride; 0.1% of body weight) mixed thoroughly with 1 L of the warm glucose solution was administered into the abomasum in the morning and evening [5] for 5 consecutive days.

### 2.3. Examination of Eggs in Feces

Fecal samples were taken directly from the rectum of the calves every morning 2 or 3 days before MCFA administration, 5 days during administration, and 2 days after the administration. Eggs of *Nematodirus* sp. in the fresh feces were quantified and examined for morphological anomalies by the sucrose flotation method [5], and eggs from the other nematodes and coccidial oocysts were also quantified. The numbers of eggs (eggs/g feces; EPG) and coccidial oocysts (oocysts/g feces; OPG) obtained over the 7-day period during and after MCFA administration were averaged and compared with those obtained before MCFA administration by paired *t*-test.

## 3. Results and Discussion

 Although calves developed diarrhea and soft feces over the course of the experiment, this was more likely due to moist herbage in the pasture than due to slight infestation with gut parasites. Identification of *Nematodirus* sp. eggs in feces was performed with ease under a light microscope, primarily by the characteristic size of the eggs [10]; the eggs of other nematode were not identified in species.

### 3.1. Egg and Oocyst Counts in Feces

Fecal shedding of eggs and oocysts before and after MCFA administration is shown in Table 1. Although one calf showed decreased fecal shedding for all nematodes, no significant difference was observed in the fecal egg counts (EPG) of *Nematodirus* sp. and other nematode taxa in response to MCFA administration. Thus, MCFA had no apparent effect on the number of enteric nematodes. While oocysts of *Eimeria bovis, E. alabamensis,* and other coccidia were observed in the feces, the OPG data shown in Table 1 refer to total numbers of oocysts. Shedding of coccidial oocysts decreased significantly after MCFA administration (*P* < .05), which is the same as reported previously [5, 6].

In all calves, MCFA administration resulted in the appearance of anomalous *Nematodirus* sp. eggs. Frequencies of anomalous egg shedding are also given in Table 1. The number of anomalous eggs that were shed after MCFA administration was smaller than the number of normal eggs in feces. However, eggs that were either severely damaged or eggs that exhibited slight degeneration may have escaped detection under the microscope. The anomalous characteristics of *Nematodirus* sp. eggs manifested as either as abnormal cell cleavage or degeneration.

### 3.2. Abnormal Egg Cell Cleavage

**Figure 1:**
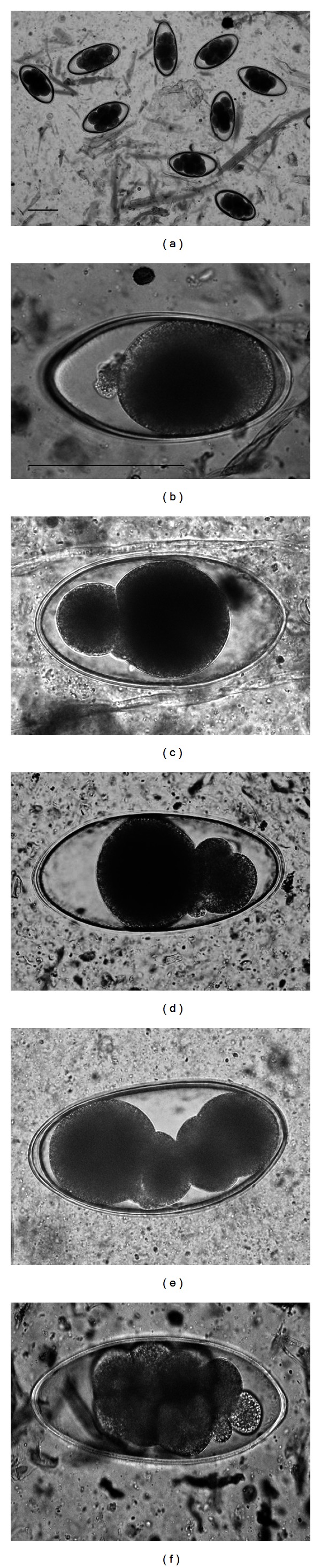
Anomalous cleavage in eggs of *Nematodirus* sp. after MCFA administration. Bar is 100 *μ*m; bar size in (b) is same as in (c–f). (a) normal, (b) abnormal monocellular egg with extracellular cytoplasmic mass, (c–f) disproportional cleavage.

Cell cleavage anomalies in *Nematodirus* sp. eggs are shown in Figure 1. In addition to frequent shedding of monocellular eggs (1-cell stage), abnormally developed eggs consisting of one cell with an additional cytoplasmic mass were shed (Figure 1(b)). Disproportional cleavage was observed in shed eggs with 2, 4, 8, and 16 cells (Figure 1(c) to Figure 1(f); 8-celled egg is not shown in Figure 1). In cattle, parasitic *Nematodirus* sp. generally shed 8- or 16-celled eggs in fresh feces [10]. By MCFA administration, the anomalies at earlier stages of egg development in the nematode ovary or in the host intestine cannot be disregarded. The effect of MCFA or its metabolic products on causing disproportional cleavage (e.g., cells within the individual egg showing different diameters) in the nematode ovary may arise in a variety of ways, but the precise mechanism is not known at present. To resolve the effect of MCFA on disproportional cleavage, future studies will need to be conducted in adult nematodes.

### 3.3. Degeneration of Eggs

**Figure 2:**
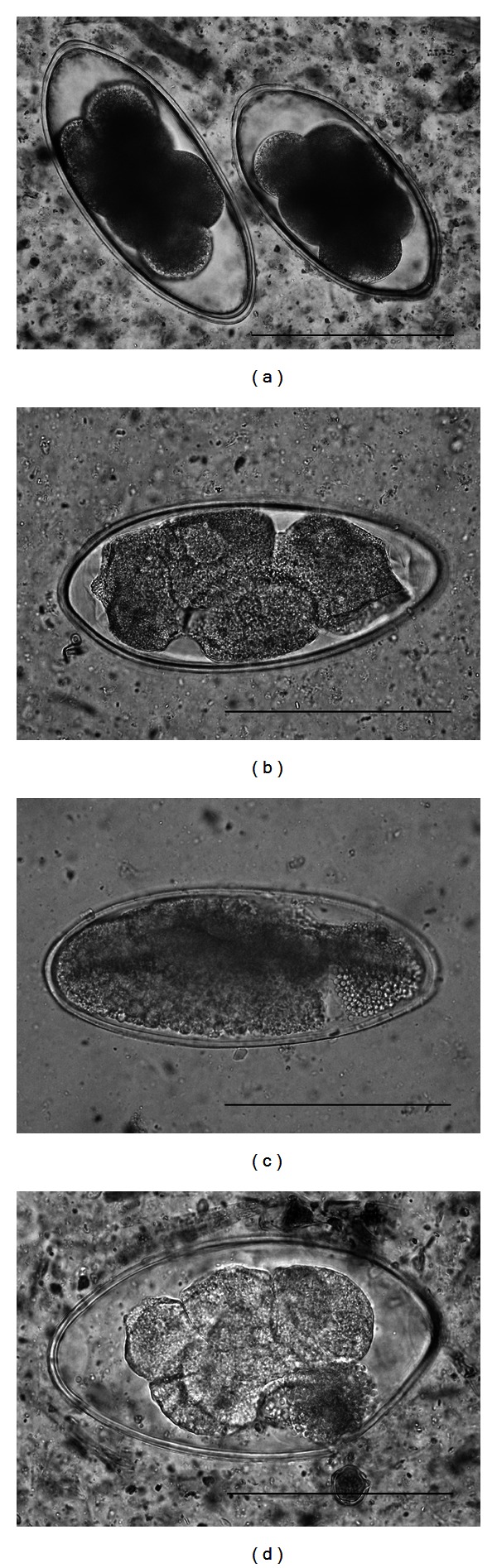
Egg degeneration in *Nematodirus* sp. after MCFA administration. Bar is 100 *μ*m. (a) normal egg, (b, c) granular and swollen degeneration, (d) outflow of fused ova content.

Degenerated *Nematodirus* sp. eggs are shown in Figure 2. After MCFA administration, degeneration of the egg manifested as granulation and swelling (Figures 2(b), 2(c)) or outflow of fused ova through shell rupture (Figure 2(d)). Not only was the degeneration restricted to the contents of the egg, but also the egg shell was affected. In addition to the strong surfactant effects of lipids generally, strong germicidal action has been attributed to MCFA [4]. However, it is important to note that these actions may vary depending on the stage of microbial growth and other environmental characteristics.

Most MCFAs retain their undissociated form in the gut lumen and can move freely across cell membranes and cause microbial lysis [1]. In addition, MCFA may also activate an autolytic enzyme (autolysin), which also lyses microbial cell membranes [4]. If autolysis begins, the egg shell might be weakened and shell rupture may occur. Lewis and Mathers [11] reported the shedding of unviable eggs showing abnormal cell cleavage and shell rupture in a parasitic nematode (*Heligmosomoides polygyrus*) from mice infected with bacteria that secreted chitinase. The degeneration of eggs reported here, particularly granulation of the ova and swelling, was similar to the degenerative changes reported in their study [11]. In the tiger puffer fish (*Takifugu rubripes*), oncomiracidia of a monogenean parasite were observed to disintegrate in incubating media containing MCFA; however, the mechanism underlying the disintegration has not been yet been clarified [8]. In addition to the aforementioned germicidal actions of MCFA, etiologic alterations of enteric flora caused by nutritional or metabolic interactions in the gut cannot be ignored. In order to better clarify the mechanism of MCFA action, studies on the adult nematode, particularly of the genital tract and *in vitro* incubation of the egg need to be undertaken in future. In the calves of the present study, although some of the eggs from the other nematodes also exhibited anomalies, specific descriptions of these findings are not given here as these eggs were not identified to species.

To conclude, MCFA administration in calves produced cleavage anomalies and degenerations in the eggs of *Nematodirus* sp. in feces. The present findings provide a new perspective on the significance of fatty acids on the embryology of gut nematodes.

## Figures and Tables

**Table 1:** Fecal shedding of parasitic eggs and coccidial oocysts before and after medium-chain fatty acid (MCFA) administration in calves.

		Before		After^†^	
		Mean	SD	Mean	SD
*Nematodirus* sp.	EPG	78	32	71	49
(Anomalous eggs)	(%)	(0)		(2.8)	(1.6)
Other nematodes	EPG	131	85	172	163
Coccidia	OPG	182	130	15*	6

(*n* = 4); EPG: Eggs/g feces; OPG, Oocysts/g feces.

^†^7-day period following MCFA administration.

*Significantly different from before MCFA administration (*P* < .05).
